# Safety of extensive pulsed field ablation for atrial fibrillation in a patient with left ventricular assist device: a case report

**DOI:** 10.1093/ehjcr/ytaf680

**Published:** 2025-12-29

**Authors:** Sana Ouali, Zeynab Jebberi, Dhekra Sebki, Manel Ben Halima, Mohamed Sami Mourali

**Affiliations:** Cardiology Department, La Rabta University Hospital, Rue Jabbari, Tunis 1007, Tunisia; Cardiology Department, La Rabta University Hospital, Rue Jabbari, Tunis 1007, Tunisia; Anaesthesiology Department, La Rabta University Hospital, Rue Jabbari, Tunis 1007, Tunisia; Cardiology Department, La Rabta University Hospital, Rue Jabbari, Tunis 1007, Tunisia; Cardiology Department, La Rabta University Hospital, Rue Jabbari, Tunis 1007, Tunisia

**Keywords:** Case report, Atrial fibrillation, Atrial flutter, Catheter ablation, Heart failure, Left ventricle assist device, Electroporation

## Abstract

**Background:**

Pulsed field ablation (PFA) is increasingly adopted for catheter atrial fibrillation (AF) ablation due to its safety and efficacy as compared to thermal energies. Left ventricular assist device (LVAD) is also an established treatment in patients with advanced heart failure. Radiofrequency catheter ablation of AF in LVAD carriers has only been case-reported. Pulsed field energy using a pentaspline catheter has never been reported for combined AF and typical atrial flutter in a continuous-flow LVAD patient.

**Case summary:**

We report the case of a 57-year-old male with advanced heart failure due to a non-ischaemic dilated cardiomyopathy. A LVAD was successfully implanted. Despite amiodarone and beta-blockers, the patient experienced symptomatic recurrent AF and atrial flutter with multiple episodes per day of low flow alarm by the LVAD. The patient was scheduled for catheter ablation and pulsed field energy was selected to shorten the time of the general anaesthesia. Under fluoroscopy guidance pulmonary vein isolation (PVI), posterior wall isolation (PWI) and cavotricuspid isthmus (CTI) ablation were successfully performed, leading to sinus rhythm restoration and symptom relief without any interference with the LVAD. At 8 months of follow-up, the patient was in sinus rhythm and did not experience any heart failure decompensation or atrial arrhythmia recurrence.

**Discussion:**

This case highlights the feasibility and the safety of PFA-assisted PVI, PWI, and CTI ablation in LVAD patients. No complications related to the transseptal approach or interference of pulsed field energy with the LVAD have been detected.

Learning points

- Pulsed field ablation catheter ablation for atrial fibrillation and atrial flutter is efficient and safe in left ventricular assist device patients
- There was no interaction between the pulsed electric field and the left ventricular assist device functioning during atrial fibrillation and atrial flutter catheter ablation.

## Introduction

Pulsed field ablation (PFA) is increasingly adopted for catheter ablation of atrial fibrillation (AF) due to its safety and efficacy as compared to thermal catheter ablation.^1^ The short duration of the procedure also offers a practical advantage, particularly in complex or high-risk cases.

Clinical trials and observational studies leading to the adoption of the PFA excluded patients with implantable cardiac devices.^2,3^ The main concern is the potential risk of interference, especially with ventricular assist devices.

Few cases of PFA have been reported in patients with continuous-flow left ventricular assist devices (LVAD), for either ventricular arrhythmia ablation^4^ or pulmonary vein isolation (PVI).^5^

We present a case report about extensive PFA catheter ablation for combined AF and atrial flutter in patients with continuous-flow LVAD.

## Summary figure

**Figure:**
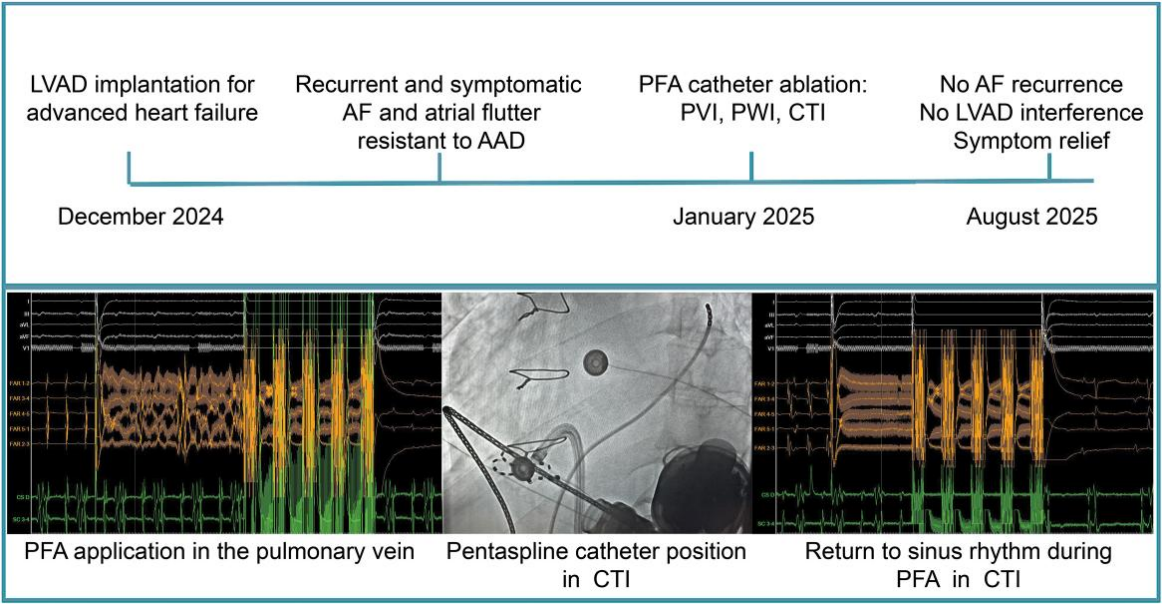


## Case presentation

**Figure 1:**
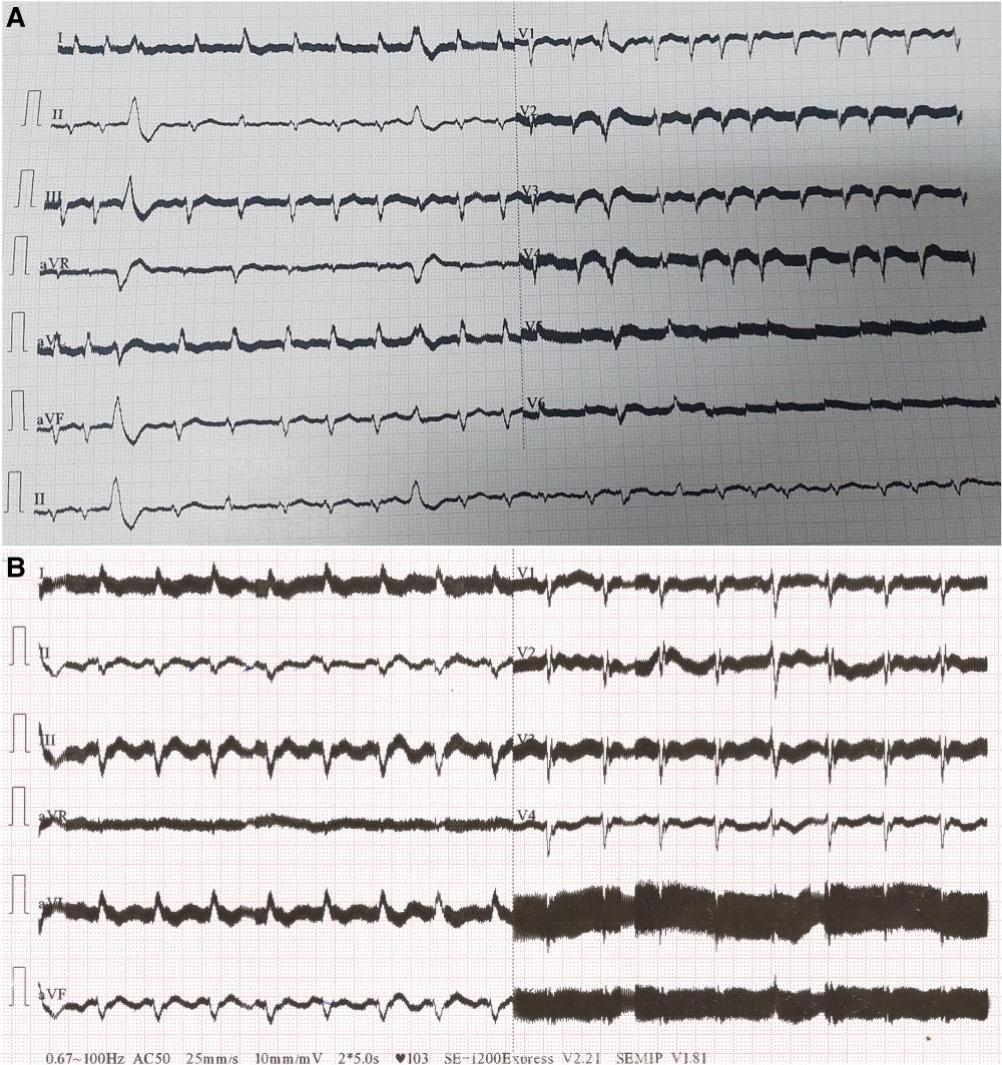
Twelve-lead electrocardiogram (ECG) showing atrial fibrillation (*A*) and typical atrial flutter (*B*). High-frequency noise artefacts were related to the electromagnetic interference generated by the left ventricular assist device.

We report the case of a 57-year-old male with a history of advanced heart failure due to a non-ischaemic dilated cardiomyopathy related to post-SARSCov2 infection myocarditis. As part of his management, LVAD (HeartMate 3 LVAD, Abbott, IL, USA) was implanted. New-onset AF has been recorded (*Figure 1A*) in the early post-operative period, leading to frequent low-flow alarm on the LVAD. Rhythm control was successfully achieved with amiodarone. One month later, frequent and recurrent daily episodes of low-flow alarm by the LVAD were recorded and all were concomitant to a typical atrial flutter at 190 b.p.m. despite treatment with beta-blockers and amiodarone (*Figure 1B*). The patient was symptomatic, expressing fatigability and asthenia.

Extensive AF ablation procedure was decided after a multidisciplinary team discussion. The approach was to perform the procedure under general anaesthesia, in accordance with hospital policy. The ablation procedure would include PVI, posterior wall isolation (PWI), and cavotricuspid isthmus (CTI) ablation, given the coexistence of poorly tolerated persistent AF and typical atrial flutter. The patient was scheduled for the PFA to shorten the time of the general anaesthesia. Transoesophageal echocardiography was performed to eliminate atrial thrombus. Anticoagulation was achieved with acenocoumarol (INR 2.6) for LVAD management.

With informed consent, the catheter ablation procedure was performed using the FARAPULSE (Boston Scientific Corporate; Massachusetts; USA) PFA system.

The patient presented to the operating room in a clockwise typical atrial flutter, without discontinuation of oral anticoagulant therapy. After transseptal puncture under fluoroscopy guidance, an intravenous heparin bolus was administered, and anticoagulation was maintained with targeted activating clotting time (ACT) of 300–400 s throughout the procedure.

**Figure 2:**
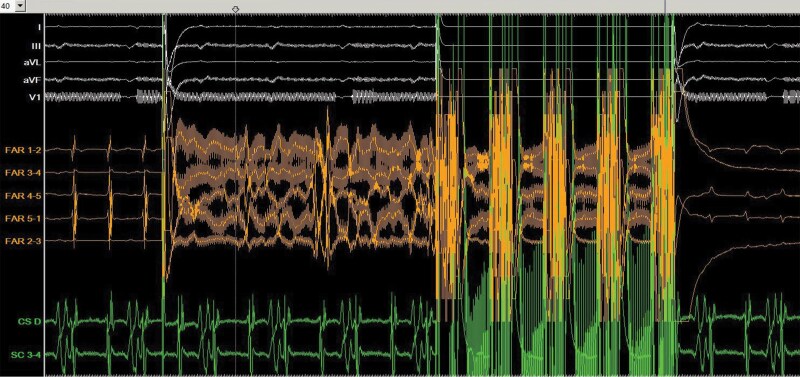
Intracardiac tracings during pulmonary vein pulsed filed ablation. The patient was in typical atrial flutter. From top to bottom: ECG tracings of lead I, lead III, aVL, aVF, and V1. Intracardiac electrogram from the Farawave catheter (FAR1-2; FAR3-4; FAR4-5; FAR 5-1; FAR2-3). Intracardiac electrogram from the coronary sinus catheter (CS distal; SC3-4).

**Figure 3:**
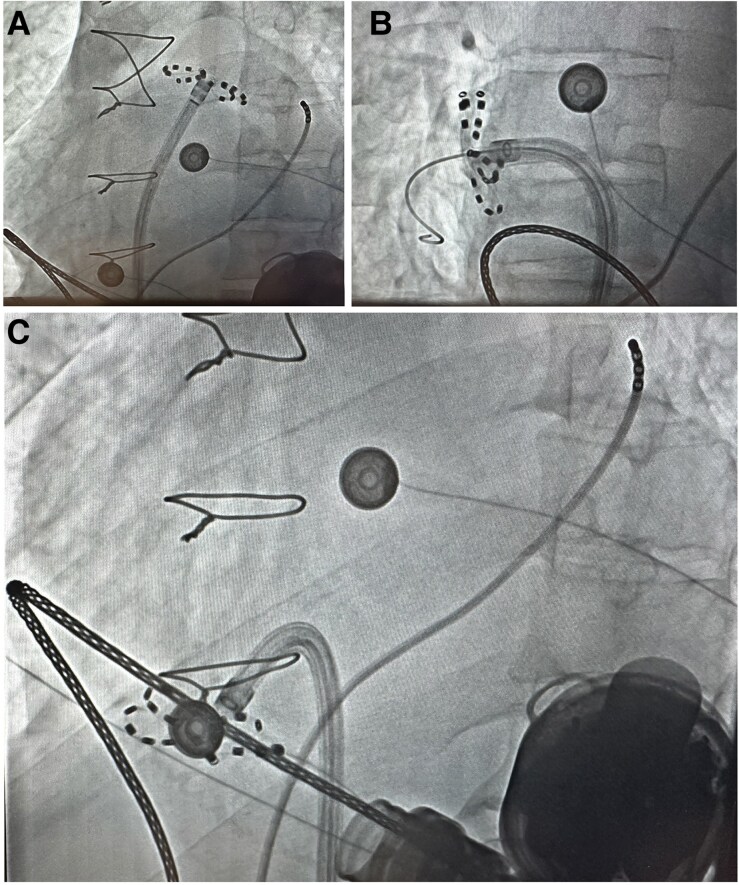
Fluoroscopic images of the pentaspline catheter during pulsed field ablation applications in flower shape configuration in the left superior pulmonary vein in left anterior oblique view (*A*), right superior pulmonary vein in right anterior oblique view (*B*), and during cavotricuspid isthmus ablation in left anterior oblique view (*C*). The quadripolar catheter is in the coronary sinus.

Pulmonary vein isolation was first achieved with the 4 × 4 approach (Farawave Basket and flower-like configurations of the catheter) (*Figures 2* and *3*). Secondly, the posterior wall ablation was performed with only the flower-like configuration of the catheter positioned along the posterior wall with overlapping lesions. Three-dimensional mapping was not performed.

**Figure 4:**
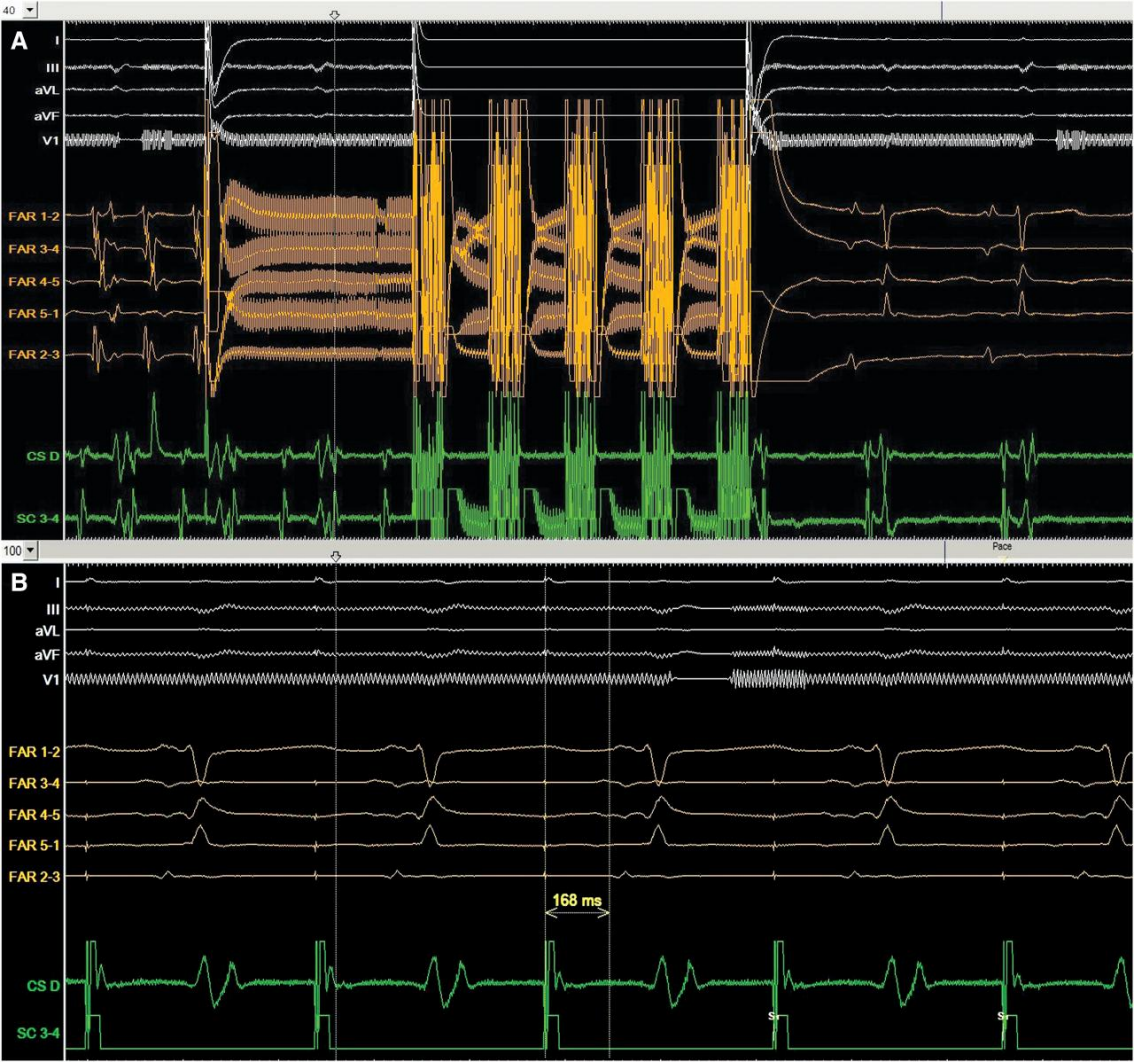
Return to sinus rhythm during pulsed field ablation in the cavotricuspid isthmus (*A*). Wide splitting of the local double potentials (168 ms) is seen in the recording from the Farawave catheter, suggesting conduction block of the cavotricuspid isthmus. From top to bottom: ECG tracings of lead I, aVL, lead III, aVF, and V1. Intracardiac electrogram from the Farawave catheter (FAR1-2; FAR3-4; FAR4-5; FAR 5-1; FAR2-3). Intracardiac electrogram from the coronary sinus catheter (CS distal; SC3-4).

Finally, after nitroglycerin administration, the CTI ablation was performed and the return to sinus rhythm was achieved within the first application (*Figure 4A*). The pentaspline configuration used was the flower-like shape (*Figure 3C*). No ST elevation neither hypotension was noted during the applications. Cavotricuspid bidirectional conduction block (*Figure 4B*) was obtained after four PFA applications and confirmed with differential pacing manoeuvers. Exit block was also confirmed in the four isolated pulmonary veins after CTI ablation.

In total, 70 PF lesions have been applied for the extensive AF ablation.

During the procedure, the flow in the LVAD ranged from 2.3 to 2.5 L/min. No short circuit leading to arcing for the PFA system or electromagnetic interference with the LVAD was noted. Left ventricular assist device interrogation did not reveal any interference.

Post procedure haemolysis was excluded within the first 48 h, as the haemoglobin value was stable as well as the haptoglobin levels. No evidence of vascular access complications or pericardial effusion was observed. The patient returned home under his usual treatment for heart failure and oral anticoagulation.

No episodes of low flow were noted since his discharge. At 8 months of follow-up, the patient was in sinus rhythm and did not experience any acute heart failure decompensation or recurrence of AF. The average flow of the LVAD was 3.5 L/min.

## Discussion

We reported a case of extensive PFA for AF and typical atrial flutter in a patient with continuous-flow LVAD. Pulmonary vein isolation, PWI, and CTI ablation were safely and successfully performed using a pentaspline PFA catheter without any electromagnetic interference with the LVAD. The standard approach in our department, for persistent AF in patients with advanced heart failure stage, is performing both PVI and the PWI. The CTI was ablated as the patient already had typical atrial flutter.

Recently, Malinowski *et al.*^5^ have described a successful and safe PVI using Farapulse PFA System (Boston Scientific) in a 48-year-old man with continuous-flow LVAD, heparin-induced thrombocytopenia and poorly tolerated persistent AF. A total of 48 PFA applications were delivered. No thromboembolic complications or electromagnetic interference were observed.

Rattka *et al.*^6^ have reported the case of a 79-year-old male with ischaemic cardiomyopathy and advanced heart failure, admitted with cardiogenic shock and alternating AF and atrial flutter. Pulmonary vein isolation and PWI using PFA, supported by the Impella CP device for mechanical circulatory support, were successfully performed, achieving sinus rhythm control and haemodynamic stability. Energy delivery at the pulmonary veins and posterior wall did not interfere with Impella function.

A recent clinical statement based on clinical observations and expert consensus postulated that catheter ablation of highly symptomatic AF/atrial tachycardia after failure of rhythm control with anti-arrhythmic drugs may be appropriate to perform in LVAD carriers.^7^ Radiofrequency was the unique energy used in the reported observations of AF catheter ablation in LVAD patients.^8,9^

The potential risk of intracardiac shunting related to transseptal approach to the left atrium was highlighted in this specific population^7^ and reserved catheter ablation to highly symptomatic patients when anti-arrhythmic drugs failed to maintain sinus rhythm.

In the present observation, no acute or midterm complications were observed including the LVAD function. Pulsed field energy was safely used in our LVAD patient despite 70 applications in the left and the right atria. Left ventricular assist device interrogation did not reveal any interference.

## Conclusion

We present a case of successful and safe use of PFA for PVI, PWI, and CTI ablation in a haemodynamically unstable LVAD patient. No electrical interference with LVAD system was detected during energy delivery. Haemodynamic stabilization, symptom relief, and sinus rhythm control were maintained during the 8-month follow-up.

## Lead author biography


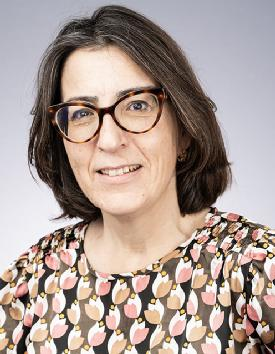


Dr Sana Ouali was born in Tunis, Tunisia. She is a professor of cardiology at the Faculty of Medicine of Tunis and Head of the Electrophysiology Unit at La Rabta University Hospital. Her research has a focus on cardiac electrophysiology in heart failure patients.

## Data Availability

The data that support the findings of this study are available from the corresponding author upon reasonable request.
